# Extraction of Lesion-Partitioned Features and Retrieval of Contrast-Enhanced Liver Images

**DOI:** 10.1155/2012/972037

**Published:** 2012-09-04

**Authors:** Mei Yu, Qianjin Feng, Wei Yang, Yang Gao, Wufan Chen

**Affiliations:** ^1^School of Biomedical Engineering, Southern Medical University, Guangzhou 510515, China; ^2^Shandong Medical College, Linyi 276000, China

## Abstract

The most critical step in grayscale medical image retrieval systems is feature extraction. Understanding the interrelatedness between the characteristics of lesion images and corresponding imaging features is crucial for image training, as well as for features extraction. A feature-extraction algorithm is developed based on different imaging properties of lesions and on the discrepancy in density between the lesions and their surrounding normal liver tissues in triple-phase contrast-enhanced computed tomographic (CT) scans. The algorithm includes mainly two processes: (1) distance transformation, which is used to divide the lesion into distinct regions and represents the spatial structure distribution and (2) representation using bag of visual words (BoW) based on regions. The evaluation of this system based on the proposed feature extraction algorithm shows excellent retrieval results for three types of liver lesions visible on triple-phase scans CT images. The results of the proposed feature extraction algorithm show that although single-phase scans achieve the average precision of 81.9%, 80.8%, and 70.2%, dual- and triple-phase scans achieve 86.3% and 88.0%.

## 1. Introduction

Computed tomographic (CT) is a primary imaging technique for the detection and characterization of focal liver lesions. Currently, CT is widely used for the diagnosis of liver tumors. A vast amount of information can be obtained from CT; however, even experienced radiologists or physicians have difficulty interpreting all the images in a certain cases within short duration. Moreover, the interpretation among radiologists shows substantial variation [1, 2], and its accuracy varies widely given the increasing number of images [3]. 

Studies on CT images retrieval have precedents [4]; however, existing medical image processing technologies are not sufficiently mature. Thus, diagnostic results are often less than ideal. Nationally, along with the developments in image processing and artificial intelligence, designing and developing systems for computer-aided diagnosis to characterize liver lesions have received considerable attention over the past years, because these systems can provide diagnostic assistance to clinicians for the improvement of diagnosis [5, 6]. Organically combining the key technologies of image processing and medical imaging has become a main research goal to provide scientific, convenient, and accurate medical means and to support diagnostic recommendations for radiologists. Such systems are implemented by image retrieval systems that enable radiologists to search for radiology patients in database and return the cases that are similar in terms of shared imaging features with their current cases. Currently, many image retrieval applications are used in the medical field. These applications are not only capable of retrieval of similar anatomical region [7–9], but also of similar lesions [10–13]. 

The most critical step in image retrieval systems is feature extraction, especially for grayscale medical images. Although low-level features, such as gray, texture, and shape [14, 15] are commonly used for visual perception of radiologic images, they cannot express the image or distinguish lesions adequately. Unfortunately, clinical diagnostic decisions are generally made based on medical imaging behavior of lesions. Therefore, the understanding of interrelatedness among the characteristics of lesion images and corresponding imaging features is critical for image training [16] as well as for features extraction. Several radiological studies have recently reported the relationship among the correlations [17]. 

The aims of the current study are three. (1) A feature extraction algorithm of hepatic lesions is provided considering the views of radiologists concerning diagnosis in triple-phase CT; (2) a content-based image retrieval (CBIR) system is developed. This system can facilitate the retrieval of radiology images that the lesions have with similar appearing to the query patient, and (3) a basis evaluation of this system is implemented. Hepatocellular carcinoma (HCC), hemangiomas, and cysts are the most common malignant and benign liver tumors. The proposed feature algorithm is derived from distinct imaging characteristics of lesion images and the surrounding liver parenchyma in triple-phase CT images, which comprise the diagnosis perspective of clinicians or radiologists for three types of tumor patients.

## 2. Methods

**Figure 1:**
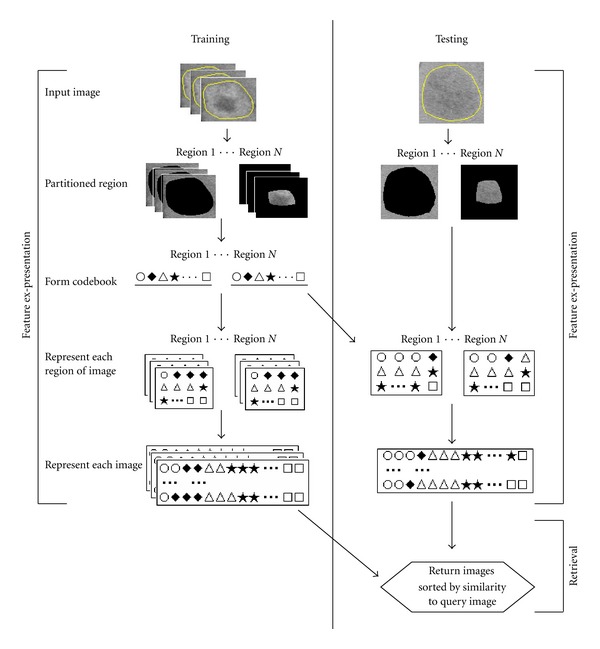
Flow chart of system based on our algorithm.

Figure 1 presents a summary of the current system based on the proposed feature extraction. The specific development and implementation are detailed below.

### 2.1. Liver Lesions

**Figure 2:**
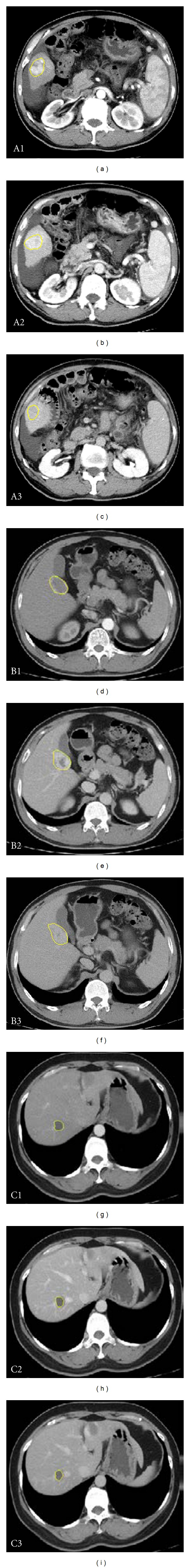
Triple-phase contrast-enhanced CT images. The row is liver cancers, hemangiomas, cysts. The vertical column is arterial phase, portal venous, and delayed-phase scans.

 Triple-phase contrast-enhanced CT scans play an important role in the diagnosis of liver tumors, because triple-phase images fully display the characteristics of blood supply richly of HCC (Figure 2). In the arterial phase, most of lesions with rich blood supply appear hyper-enhancement. Density is significantly higher than that of normal hepatic parenchyma, because hepatic parenchyma has not reached the enhanced peak. In the portal venous phase, the parenchyma reaches its peak, whereas the lesion almost joins the blood supply. The tumor is characterized by low-density nodules relative to parenchyma. “Fast in and fast out” is the most characteristic movement of HCC with rich blood supply. 

The CT scan is the preferred imaging methods for hepatic hemangiomas. The enhanced characteristic of a hemangioma is as follows. The edge of the lesion in the arterial phase usually appears heavily enhanced, and the contrast agent gradually enters the lesion, traveling from the edge to the centre over time, which provides a reliable basis for diagnosing HCC and hemangioma. “Fast in and slow out” is the most characteristic movement for hemangiomas. Therefore, the density of the lesion is higher than that of parenchyma in the arterial phase and is lower than that of parenchyma in the portal venous lesion, which is also the typical behavior that distinguishes HCC and hemangiomas.

Liver cysts are commonly benign, and triple-phase enhanced CT scans of such cysts appear as single or multiple, round or oval, and with a smooth edge and uniform low density. The value of CT is close to water. Images of liver cysts are subjected to no further enhancement after contrast enhancement.

Two facts summarize the characteristics of triple-phase contrast-enhanced CT images. First, most lesions of HCC and hepatic hemangiomas have special characteristic changes, whereas no change occurs in that of cysts. Second, the surrounding liver parenchyma information of a lesion is important because of the discrepancy in density between the lesion and the adjacent normal parenchyma in triple-phase scans. Thus, according to the above analysis, a feature extraction algorithm of lesions is proposed considering the specific behavior of focal liver lesions and their surrounding liver parenchyma after enhancement.

### 2.2. Computer-Generated Features

*Representation of Bag of Visual Words Combined with Distance Transformation*. The proposed feature extraction algorithm aims to meet the requirements of radiologic diagnosis views, as derived formed from the analysis in Section 2.1. The algorithm includes mainly two processes: (1) distance transformation, which is used to divide the lesion into distinct regions and represents the spatial structure distribution and (2) the representation of bag of visual words (BoW) based on regions, which is the key step. Generally, the lesion is divided into three regions in the experiments, to best fit the imaging analysis of radiologists in triple phases for the diseases described above. The effect of number selection on the performance of CBIR is discussed later in this document. The algorithm is described below.

#### 2.2.1. Partition of the Lesion through Distance Transformation

The concept of distance transformation has been widely used in image analysis, computer vision, and pattern recognition since its introduction by Rosenfeld and Pfaltz [18] in 1966. 

Distance transformation is conducted against binary images to produce a grayscale image, such as the distance image. The gray values of every pixel point in the distance image are the distances between the pixel and its nearest background pixels. In two-dimensional space, a binary image contains only two kinds of pixels: target pixels and background pixels. The value of a target pixel is 1, and the value of a background pixel is 0. Currently, a variety of distance transformation algorithms is used, and these algorithms adopt mainly two types of distance: non-Euclidean distance and Euclidean distance. The former method commonly includes city-block, chessboard, and chamfer. City block distance transformation is used in this paper because the distance value after transformation is an integer, which is more convenient for the subsequent partition of lesions. 

Then, the distance transformation image of the binary image is obtained. Set *p*, *q* as the quotient and the remainder, respectively, resulting from the division of the number of layers by three. Divide the lesion into three regions, and the number of layers in each region become *p*, *q* and *p* + *q*, respectively, from the boundary of the lesion to the center.

**Figure 3:**
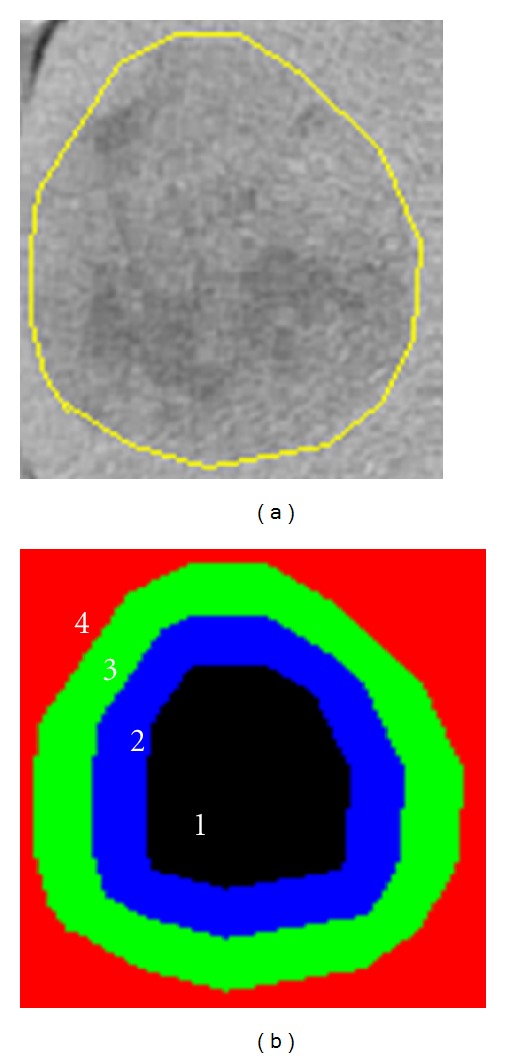
Partition of hepatic lesion. (a) is the lesion with external neighborhood and (b) is 4 regions divided.

Apart from considering the lesion changes, the density discrepancy between the adjacent normal liver parenchyma and the lesion in triple phase scans is also a foundation for the diagnosis of radiologists. Therefore, the surrounding liver parenchyma of lesions is considered as the fourth region (Figure 3). Assuming the bounding box of the lesion region is *K*′ × *L*′ if each side of the box has an extension of two pixels, a bounding box that includes the lesion with size (*K*′ + 4) × (*L*′ + 4) is finally obtained. Thus, the new box not only contains the tumor, but also its surrounding normal liver parenchyma. 

#### 2.2.2. Regional BoW

 Typically, BoW representation involves four major steps: (1) patches of interest image regions are detected; (2) patches are locally described using feature vectors (local descriptor); (3) features are quantized and labeled in terms of a predefined dictionary, that is, the construct process of codebook; (4) histograms are constructed by accumulating the labels of the feature vectors of each image in database.

In the following experiments, the BoW approach, used generally, follows the traditional visual codebook method [19–22]. The approach is accomplished by selecting patches from images, characterizing them with the vectors of the local visual descriptors, and labeling the vectors using a learned visual codebook. The occurrence of each label is quantified to build a global histogram that summarizes the image content. The histogram is then subjected to distance metric methods to estimate the disease category label of the images. The patches are extracted from each pixel point of the tumor images. The codeword vocabulary is typically obtained by clustering the descriptors of the training images. The intensity values are adopted to characterize the local visual descriptors, which implicitly reflect the category of the lesion in CT images, thereby providing more important information. Unsupervised *K*-means clustering is chosen as the base codebook learner in the current paper.

The selected size of square patches is seven. This selection considers the limitation of too many images in the database and the size restriction of lesions like cysts. Basically, an image is represented by a histogram of word frequencies that describes the probability density over the code words in the codebook. The background gray value of a radiologic image is 0. Thus, the patches that contained more background pixels are removed to save time and simply computational. The number of pixels with a value in patch not equal to 0 is set to be greater than 15 in this paper. For the vocabulary *V* = {*v*_1_, *v*_2_,…, *v*_*N*_} with *N* code words, the traditional codebook model estimated the distribution of the code words in an image of *r* patches {*p*_1_, *p*_2_,…, *p*_*r*_} by *x* = [*x*_1_, *x*_2_,…, *x*_*N*_]^*T*^, where

(1) $$\begin{array}{c} x_{i}=\sum_{i=1}^{r}\{\begin{array}{cc} 1 & \text{if}\;v_{i}=\arg\underset{u\in V}{\min}\mathrm{dist}\left(u,p_{k}\right),\\ 0, & \text{otherwise}, \end{array} \end{array}$$

denoting dist⁡(*μ*, *p*_*k*_) to be the distance between code word *μ* in vocabulary and image patch.

**Figure 4:**
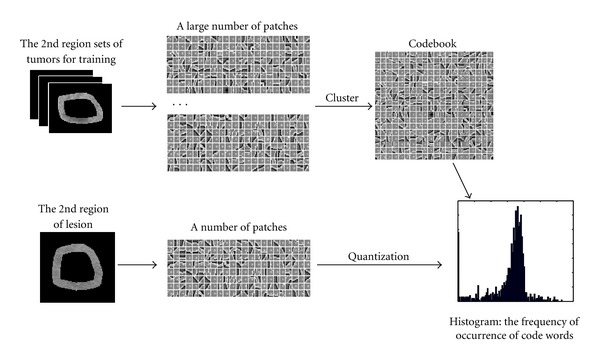
The BOW feature extraction based on regions of lesion.

The quantized vectors of each region of the lesion are obtained using the method described above. The final feature of the lesion is expressed as the arrangement. The process of BoW is shown in Figure 4. The features of each patient in different phases are calculated to determine the average of all corresponding images. For example, if an HCC patient has six images (one arterial phase image, three portal venous phase images, and two delayed phase images), the features used in retrieval of the portal venous phase, dual-phase (arterial phase plus portal venous phase), or triple-phase are the corresponding average of features of three images, four images, or all six images.

### 2.3. Common Low-Level Features

 For each lesion, multiple features are computed within the lesion region of interest (ROI).

#### 2.3.1. Intensity Features

The following five intensity features are calculated: mean, standard deviation, entropy, skewness, and kurtness of gray-level histogram [23].

#### 2.3.2. Texture Features

 Gray-level cooccurrence matrix (GLCM) [23–25] and Gabor [26, 27] describe the texture characteristics of each ROI. Sixteen GLCM features are calculated in the current experiments using contrast, homogeneity, energy, correlation for four angles (i.e., 0°, 45°, 90°, 135°), and distance of 1. Next, 48 Gabor features are computed from the mean and standard deviations of the energy in the frequency domain over four scales and six orientations. The mean and standard deviations of high-frequency coefficients of its three-level Daubechies4 wavelet decomposition are computed, resulting in 12 features.

#### 2.3.3. Shape Features

The statistics of wavelet coefficients of the shape signature are used to characterize the shape of tumors. The one-dimensional shape signature *S*(*i*), based on radial distance, is defined as follows:

(2) $$\begin{array}{c} S\left(i\right)=\sqrt{{\left(x\left(i\right)-C_{x}\right)}^{2}+{\left(y\left(i\right)-C_{y}\right)}^{2}}, \end{array}$$

where *x*(*i*) and *y*(*i*) are the coordinates of the *i*th point on the tumor boundary and *c*_*x*_ and *c*_*y*_ are the coordinates of the centroid of the tumor region. Twelve features are computed from the mean and variance of the absolute values of the wavelet coefficients in each subband by the five-level one-dimensional wavelet decomposition.

This computation yields a total of 93 features.

### 2.4. Similarity Distance Measure

When the feature vectors containing detailed imaging information of lesions are computed, the system calculates similar distance measures between them, that is, similarity between the corresponding images. The similarity of lesions is defined as the distance between the corresponding elements of the respective feature vectors that describe the lesions. Previous studies have shown that well-designed distance metrics can result in better retrieval or classification performance compared with Euclidean distance [28–31]. The goal of distance metric learning is to determine a linear transformation matrix to project the features into a new feature space that can optimize a predefined objective function. The distance metric learning algorithms used in this paper are L1 distance, L2 distance, regularized linear discriminant analysis (RLDA) [32–34], and linear discriminant projections (LDP) [35–37]. 

The distance in the L1 norm is known as Manhattan distance. The L2 norm distance is called the familiar Euclidean distance. The L1 and L2 distance are described as follows:

(3) $$\begin{array}{c} \text{Distance-L}1\left[x^{l},x^{q}\right]=\sum_{i=1}^{N}\left|x_{i}^{l}-x_{i}^{q}\right|,\\ \text{Distance-L}2\left[x^{l},x^{q}\right]={\left(\sum_{i=1}^{N}{\left(x_{i}^{l}-x_{i}^{q}\right)}^{2}\right)}^{1/2}, \end{array}$$

where *x* ^*q*^, *x* ^*l*^ represent the features of the query cases and the cases in the database and *N* is the dimension of features.

The RLDA was first presented in [32]. Ye and Wang then proposed an efficient algorithm to compute the solution for RLDA [33]. The performance of RLDA exceeds that of ordinary linear discriminant analysis (LDA) methods [34]. The numbers of samples is set to *M*. When *M* and the feature dimension *N* are large, applying RLDA is not feasible because of the memory limit. Considering *N* is large in the current paper, the dimension is first reduced to *M* − 1 using principal component analysis (PCA), after which RLDA is applied. Parameter *α* controls the smoothness of the estimator in RLDA. The value of *α* is set to 0.001.

The details of the LDP have been inferred from the previous papers. The LDP approach has three advantages. First, LDP can be adapted to any dataset and any descriptor, and may be directly applied to the descriptors. Second, LDP is not sensitive to noise and is thus suitable for work on hepatic CT images. Third, LDP can be trained much faster because the *k*-nearest of each sample point does not need to be determined [37]. Moreover, LDP has been proven to produce better results than some other approaches.

### 2.5. Lesion Database

All the imaging data of hepatic CT images for experiments were acquired from the General Hospital of Tianjin Medical University between February 2008 and October 2010. CT examinations were performed with a 64-detector helical scanner (LightSpeed VCT; GE Medical Systems, Waukesha, Wis). The following parameters were used: 120 kVp, 200–400 mAs, 2.5–5 mm section thickness, and a spatial resolution of 512 × 512 pixels. The imaging data included three diseases: HCC, hemangiomas, and cysts. In all, 1248 DICOM lesion images (498 HCC, 481 hemangiomas, and 269 cysts) were found in 187 patients (89 HCC, 54 hemangiomas, and 44 cysts) wherein each patient corresponded to 2–10 images. All images were classified into arterial phase, portal venous phase, and delayed phase, wherein the number is 388, 443, and 417, respectively. All the images in the database were manually delineated using semiautomatic segmentation to ensure the effectiveness of the CBIR system, and some inaccurate results were reevaluated by medical imaging experts blinded to the final diagnosis to obtain more precise lesion data. 

In the current study, a patient is a query case. Each patient has more than one lesion. For example, he/she may have some cysts or have got hemangioma as well as cysts. However, only one typical lesion is selected from each image of each patient, and the lesions in each patient are the same. All the images of each patient are used for query. The features of the patient are the average value of features of the images in single-, dual-, and triple-phase scans.

### 2.6. Evaluation Measures

 Precision and recall are common criteria used in evaluating the effectiveness of CBIR. Precision indicates the accuracy of retrieval, that is, how exclusively the relevant images are retrieved. Precision and recall ratio can be defined as follows:

(4) $$\begin{array}{c} \text{Precision}=\frac{\text{Number}\;\text{of}\;\text{relevant}\;\text{images}\;\text{retrieved}}{\text{Total}\;\text{number}\;\text{of}\;\text{image}\;\text{retrieved}},\\ \text{Recall}=\frac{\text{Number}\;\text{of}\;\text{relevant}\;\text{images}\;\text{retrieved}}{\text{Total}\;\text{number}\;\text{of}\;\text{relevant}\;\text{image}}. \end{array}$$

The higher the value of these two indicators, the better the retrieval system. The two indicators are usually mutually contradictory. In theory, as precision increases, recall decreases, and vice versa. Therefore, generic retrieval systems that optimally balance these two indicators achieve better retrieval performance. Generally, the ultimate goal of the proposed CBIR system is to achieve retrieval results that better reflect the actual categories of the query case. The CBIR system retrieves similar cases and thereby calculates a decision value (i.e., similar distance) that describes the similarity to the query case. Therefore, precision is needed. The higher the precision, the more relevant cases are retrieved, which indicates that the CBIR system has important clinical applications. The evaluation measurement is mainly the average precision, which is defined as the average ratio of the number of relevant images returned over the total returned images. Therefore, in the current experiment the following measures are used to evaluate the CBIR system.

- Here, P(10), P(20), and P(*n*), the average precisions after the top 10, 20, and *n* patients are returned when lesion images are ranked according to similarity to a query lesion.
- Mean average precision (MAP) is the mean of the average precisions when the number of images returned is varied from 1 to the total number of images.
- Precision versus recall graph.

### 2.7. Training and Evaluation

All the experiments are based on 187 patients, using the *K*-fold cross-validation (*K*-CV) method. *K*-CV is used for the allocation of the samples. All the samples are evenly divided into *K*, where in *K*–1 samples are chosen to training, and the remainder performs the validation alternately. In this paper, the sampling plan for *K*-CV is as follows: 187 cases are evenly divided into *K*, and each is used for testing set, whereas the other *K* − 1 samples are used for training sets. Thus, an *K*-CV experiment needs to establish *K*-models, that is, perform the *K* tests. Generally in practice, the value of *K* needs to be sufficiently large to enable a sufficient number of training samples, which enables the distribution features of images in training sets to be sufficient for describing the distribution features of the entire image sets. Thus, the distribution features of images in the entire database are not significantly influenced when some images of a new patient are added. A *K* value equal to 10 is considered adequate; hence the value is set to 10 in the experiment. Each patient in each test is searched as a query case. Thus, the average precision of each test and the MAP of 10 tests are obtained. 

## 3. Experiment and Results 

The effectiveness of the proposed feature extraction method was verified by retrievals of single-, dual-, and triple-phase scans to maximize the average precision of a large hepatic CT image dataset. Four experiments were performed: (1) verification of the proposed algorithm given different conditions; (2) comparison between general low-level features and high-level BoW features, (3) exploration of the selection of region number, and (4) identification of the amount of clusters influence. PCA was used for dimension reduction because of the initial huge dimension first. Arterial phase, portal venous phase, and delayed phase are abbreviated as AP, PVP, and DP.

### 3.1. Retrieval Results of Regional BoW

**Figure 5:**
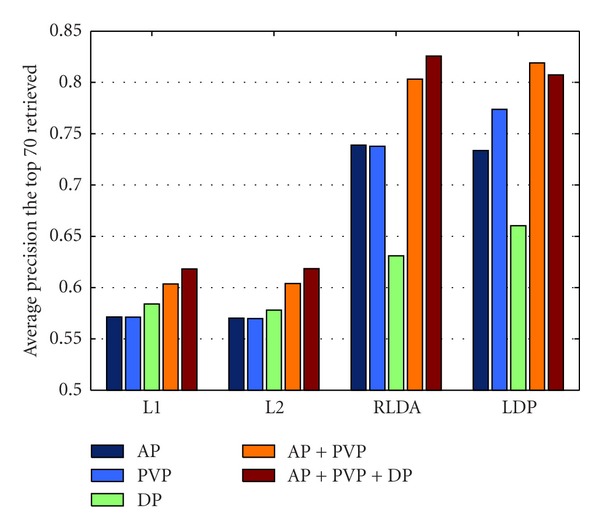
The average precision histogram of staging retrieval-based 3 regions using four distance metrics.

 The retrieval performance of the proposed feature extraction algorithm in single-, dual-, and triple-phase scans is shown in this experimental. The dual-phase not only refers to arterial plus portal venous phase, but also to portal venous plus delayed phase.  Figure 5 provides the retrieval results based on three regions of lesions using the four distance metric methods mentioned above in terms of P(70). The figure shows that the results of single-phase scan are lower than the results of two dual-phase and triple-phase scans, and that the use of RLDA and LDP generate better results than the use of L1 and L2. 

Table 1 shows the retrieval performance based on three regions, together with their surrounding liver parenchyma in triple phase scans in terms of MAP, P(10) and P(20). The estimated P(20) of single-phase scans using RLDA and LDP is lower than 85.8%, whereas the P(20) of dual-phase and triple-phase scans is higher than 91.2%, except for PVP + DP.  Figure 5 and Table 1 indicate that the dual-phase and triple-phase scans are more precise than the single-phase scans, because regional BoW-based features greatly express the characteristics of the three tumors in the triple phases, that is, HCC and hemangiomas mostly exhibit characteristic changes, whereas no change occurs in cysts. Therefore, the proposed feature extraction method agrees with the diagnosis of the radiologist for the three lesions. In short, although single-phase scans may play an important role in diagnosis or detection, dual-phase and triple-phase scans also ensured more accurate diagnosis than single-phase scans. The founding demonstrates that arterial and portal venous phase scans play a major role in diagnosis, and explains why radiologists directly diagnosed some hepatic diseases only through dual-phase scans (i.e., artery plus portal venous phase). 

**Figure 6:**
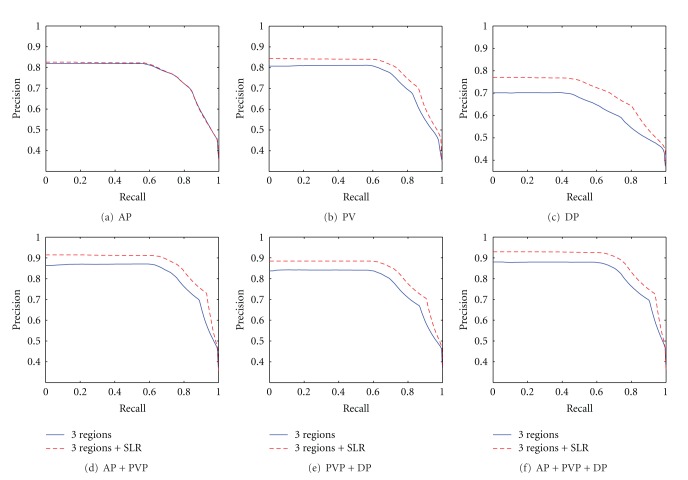
Precious versus recall curve in triple phases based on 3 Regions and 3 Regions with neighborhood, and SLR represents surrounding liver parenchyma.

Figure 6 shows the precision versus recall curves of the three regions, as well the region with their surrounding liver parenchyma in triple phases. The figure shows that the retrieval performance of the latter is better than performance of the former, regardless of single-, dual-, or triple-phases scans. Thus, the results that consider the surrounding liver of the lesion are more accurate because the discrepancy in density between the lesions and their adjacent liver parenchyma in the triple phase scans is also considered by radiologists as the basis for HCC, hemangiomas, and cysts diagnoses.

**Figure 7:**
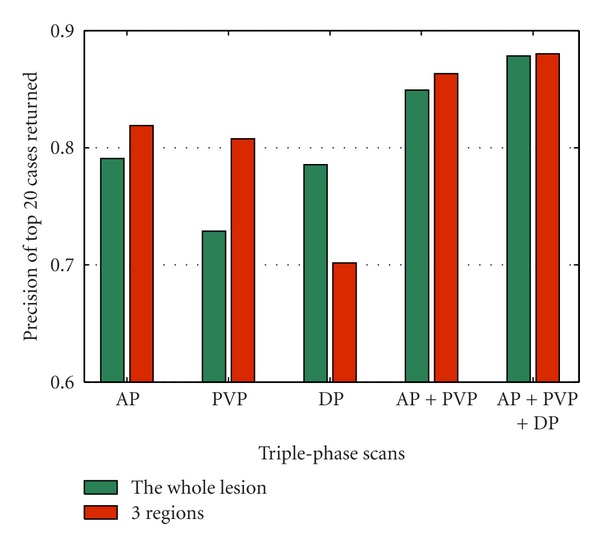
The retrieval performance comparison based on whole lesion and 3 regions.

**Figure 8:**
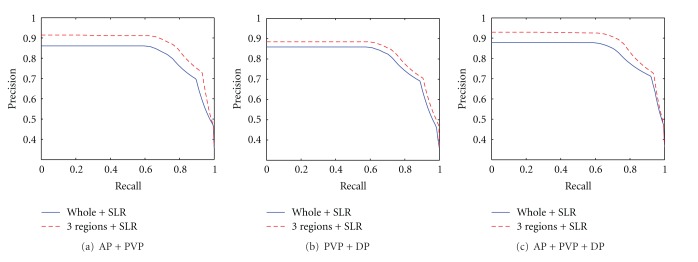
Precision-recall curve of dual-phase and three-phase scanning retrieval, and SLR represents surrounding liver parenchyma.

The validation of our proposed algorithm can be seen from different perspectives. Figures 7 and 8 separately compare the performance among three regions of lesion and the whole lesion, as well as the comparison between two cases with surrounding liver parenchyma. Figures 7 and 8 show that the results based on the regions always outperform the whole lesion with or without surrounding liver tissues in triple phases. This result can be attributed to the imaging characteristics of the enhanced images, quantitatively expressed by the proposed feature extraction algorithm. 

### 3.2. Comparison of Common Low-Level Features and High-Level BoW Features

**Figure 9:**
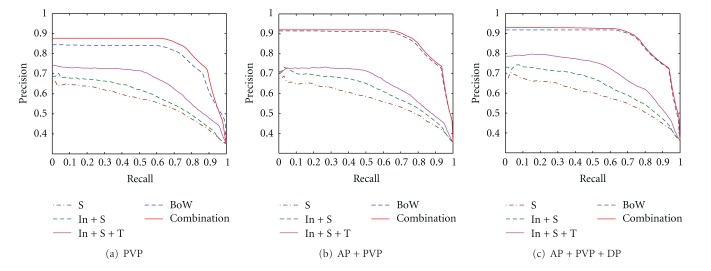
Precision-recall curve using different features in PVP, dual-phase and triple-phase scans.

Common low-level features were compared with the proposed feature extraction algorithm in terms of precision versus recall curves.  Figure 9 provides the results using shape alone (denoted as S), combination of shape and intensity (denoted as In+S), combination of shape, intensity, and texture (denoted as In+S+T), BoW alone, and combination of all features mentioned in Section 2.3 in PVP, dual-phase and triple-phase scans. As shown, the combination of intensity and shape outperforms shape alone, whereas the combination of intensity, shape, and texture yields better results than the combination of intensity and shape. BoW alone outperforms the combination of common features, whereas the combination of all features mentioned is superior to BoW alone. Notably, the CBIR system based on our algorithm is better than the system based on other different feature extraction algorithms from Figure 9 due to the fact that our proposed approach can express the imaging characteristics of lesions in triple phases.

### 3.3. Discussion of the Number of Regions for Lesions

 The number of regions that the lesion is divided into is set as parameter *s*. The effects of parameter *s* on our retrieval system are discussed in this section.  Table 2 shows the average precision after retrieving the top 20 cases with the parameter *s* from 2–5. For convenience, only the dual-phase and triple-phase scans were used. When *s* is 2 and 5, the results of all multiple phases are below 90%; when *s* is 3 and 4, the results of some multiple phases are better than 90%; when *s* equal to 3, the best retrieval performance is achieved of all the *s* values, as shown in Table 2. 

### 3.4. Influence of the Amount of Clusters

**Figure 10:**
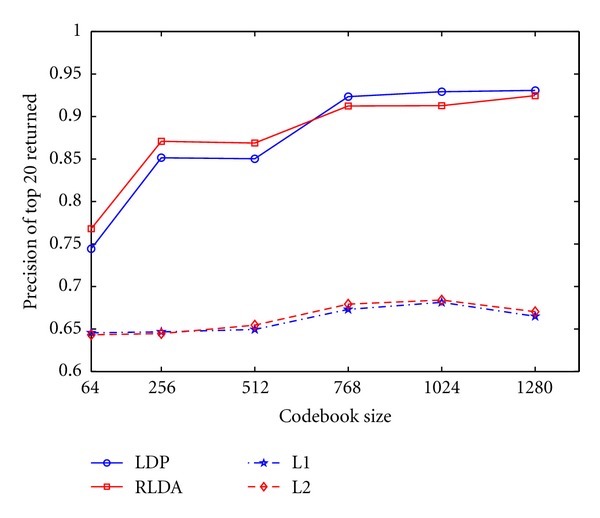
The average precision of top 20 cases returned in triple-phase scan with different dictionary sizes.

Figure 10 shows the effects of the amount of clusters (i.e., codebook size) using distance metric L1, L2, RLDA, LDP. The plots show that performance increases with the number of codebook sizes; however, a large dictionary results in high computation cost. Thus, the codebook size is set to1024 in our paper.

## 4. Conclusion

We have developed a regional BoW feature extraction algorithm for lesion images. The proposed algorithm is mainly based on the imaging characteristics of lesion visible on contrast-enhanced triple-phase CT images. Our CBIR system, which incorporates the proposed feature extraction algorithm, can practically retrieve three types of liver lesions that appear similar. The accurate assessment of our approach shows reasonable retrieval results that are in accordance with the diagnoses of radiologists. Our system can aid in decision-making related to the diagnosis of hepatic tumors and support radiologists in multiphase contrast-enhanced CT images by showing them similar patients in lesions.

## 5. Discussion

The development of a feature extraction algorithm for lesion images is presented in this paper. Our experiments show that a CBIR system incorporated with this algorithm can yield excellent retrieval results. The development of this algorithm considers the imaging characteristics of three lesions and their surrounding normal liver parenchyma in contrast-enhanced triple-phase CT images. This algorithm combines feature vectors with the characteristic of the ROI, which is very essential in retrieval systems. Thus, our algorithm is powerful and more advantageous compared with common low-level feature vectors. The system may serve as useful aided diagnosis system for inexperienced or experienced radiologists in searching databases of radiologic imaging and obtaining good retrieval results. 

A number of studies have been conducted on various hepatic tumor imaging technologies [23, 24, 26]. Mougiakakou et al. [23] defined an aided diagnosis system for normal liver, hepatic cyst, hemangioma, and HCC in nonenhanced CT scans. Zhang and his colleagues [24] used an aided diagnosis system to segment and diagnose enhanced CT and MR images of HCC. More recently, a CBIR system that closely resembles the current study was presented [26]. In this system, metastases, hemangiomas, and cysts were all visible on portal venous phase images. However, the images used common low-level features such as intensity, texture, and shape, and did not consider the imaging characteristics of lesions in multiphase scans. In our opinion, multiphasic imaging is central to current clinical diagnosis. 

In the present paper, the use of BoW was verified to be effective. Although existing image retrieval technologies achieved some good performances, they still have some limitations. Most of image retrieval technologies are based on the underlying characteristics of the images and used low-level features. Therefore, they are unable to resolve the semantic gap problem, which is the inconsistency between the low-level visual features and the high-level semantic features. BoW, as a high-level feature [38], has obtained great success in text retrieval problem, because of its speed and efficiency, and has been gaining recognition in its use in problems such as object recognition and image retrieval from large databases [22]. The BoW framework ignores the spatial configuration between visual words (i.e., the link between the characteristics and location) and can cause information loss. However, this framework can quickly and easily build a design model. In the proposed feature extraction algorithm, the spatial structure information of images is considered by dividing the lesion into three regions, which compensates for the lack of BoW. Thus, BoW successfully represents the regional features.

Semantic features are not considered in our study because of two factors. First, if the query patient remains undiagnosed, semantic features cannot be used because of lack of radiology reports. Second, radiologists may use different terminology to describe the same observation [39, 40]. Thus, some inevitable subject factors exist in the semantic annotation.

The number of regions that the lesions are divided into is set mainly due to the triple phase scans of the three tumors. The optimal number of regions is 3, as revealed in our previous some experiments, and we have verified that retrieval performance is the best of the number from 1 to 5. Dividing the lesion into three regions fits the best imaging behavior of lesion in triple phases described in Section 2.1. Thus, our selected number of regions is theoretically conducive to our proposed feature extraction algorithm.

Our retrieval system was implemented and evaluated according to the patients, namely, the images were grouped according to the patient and the patient is the primary unit of the query and retrieval. This scheme is very different from the traditional CBIR system, where single image or single slice is used as query and retrieval. This patient-based fashion is more helpful for the diagnosis aid, because the multiphase images from the retrieved patient obviously could supply more information than just one image for making decision for current query patient. The development of our feature extraction algorithm is focused on the views of imaging findings of the three lesions in triple-phase scans. Therefore, the retrieval process in our experiments follows single-, dual-, and triple-phase scans to verify the practicality of our retrieval system based on the proposed method.

Our study mainly has two main limitations. The first is number of lesions types used, which was limited to three. The proposed feature extraction algorithm was developed based on the imaging characteristics of three lesions (HCC, hemangiomas, and cysts) in contrast-enhanced triple-phase images. HCC is a common malignant tumor, whereas hemangiomas and cysts are the common benign cells. Studies on hepatic lesions and lesions in other body areas can be extended in future work to encourage continued development of relevant feature extraction methods. The second limitation is the segmentation of lesions used in our system. Lesions should first be segmented from abdominal CT images because lesions contained important imaging information for image retrieval. Several segmentation algorithms [24] have been proposed to achieve automatic or semiautomatic segmentation in medical image analysis. However, because of the complexity of medical images and lesion infiltration, no standard method can generate satisfactory segmentation results for all hepatic-enhanced images. Consequently, manual segmentation is employed by imaging experts to obtain more accurate lesion images. 

In conclusion, the CBIR system based on our proposed feature extraction algorithm has practical application in aided diagnosis, which can help radiologists to retrieve images that contain similar appearing lesions. 

## Figures and Tables

**Table 1:** Retrieval result based on 3 regions with surrounding liver parenchyma.

Staging scan	MAP		P(10)		P(20)	
	RLDA	LDP	RLDA	LDP	RLDA	LDP
AP	0.6160	0.6135	0.8259	0.8192	0.8259	0.8190
PVP	0.6288	0.6333	0.8445	0.8575	0.8445	0.8575
DP	0.5972	0.5960	0.7707	0.7723	0.7707	0.7723
AP + PVP	0.6650	0.6651	0.9146	0.9144	0.9141	0.9144
PVP + DP	0.6522	0.6536	0.8845	0.8900	0.8845	0.8900
AP + PVP + DP	0.6755	0.6681	0.9292	0.9127	0.9292	0.9123

**Table 2:** Retrieval results with different number of regions.

*s*		AP + PVP	PVP + DP	AP + PVP + DP
2	RLDA	0.8795	0.8670	0.8969
	LDP	0.8849	0.8724	0.8968
3	RLDA	0.9141	0.8845	0.9292
	LDP	0.9144	0.8900	0.9123
4	RLDA	0.9080	0.8836	0.8906
	LDP	0.9136	0.8895	0.9079
5	RLDA	0.8621	0.8443	0.8742
	LDP	0.8621	0.8382	0.8799
